# Role of circRNA-miRNA-mRNA interaction network in diabetes and its associated complications

**DOI:** 10.1016/j.omtn.2021.11.007

**Published:** 2021-11-10

**Authors:** Shukla Sakshi, Ravichandran Jayasuriya, Kumar Ganesan, Baojun Xu, Kunka Mohanram Ramkumar

**Affiliations:** 1 Department of Biotechnology, School of Bioengineering, SRM Institute of Science and Technology, Kattankulathur, 603 203 Tamil Nadu, India; 2 School of Chinese Medicine, Li Ka Shing Faculty of Medicine, The University of Hong Kong, Hong Kong, China; 3 Food Science and Technology Program, BNU-HKBU United International College, Zhuhai 519087, China

**Keywords:** circRNA-miRNA-mRNA, diabetes, diabetic complications

## Abstract

The majority of the non-protein-coding RNAs are being identified with diversified functions that participate in cellular homeostasis. The circular RNAs (circRNAs) are emerging as noncoding transcripts with a key role in the initiation and development of many physiological and pathological conditions. The advancements in high-throughput RNA sequencing and bioinformatics tools help us to identify several circRNA regulatory pathways, one of which is microRNA (miRNA)-mediated regulation. Besides the direct influence over mRNA transcription, the circRNA can also control the target's expression via sponging miRNAs or the RNA-binding proteins. Studies have demonstrated the dysregulation of the circRNA-miRNA-mRNA interaction network in the pathogenesis of many diseases, including diabetes. This intricate mechanism is associated with the pathogenesis of diabetes and its complications. This review will focus on the circRNA-miRNA-mRNA interaction network that influences the gene expression in the progression of diabetes and its associated complications.

## Graphical abstract

**Figure:**
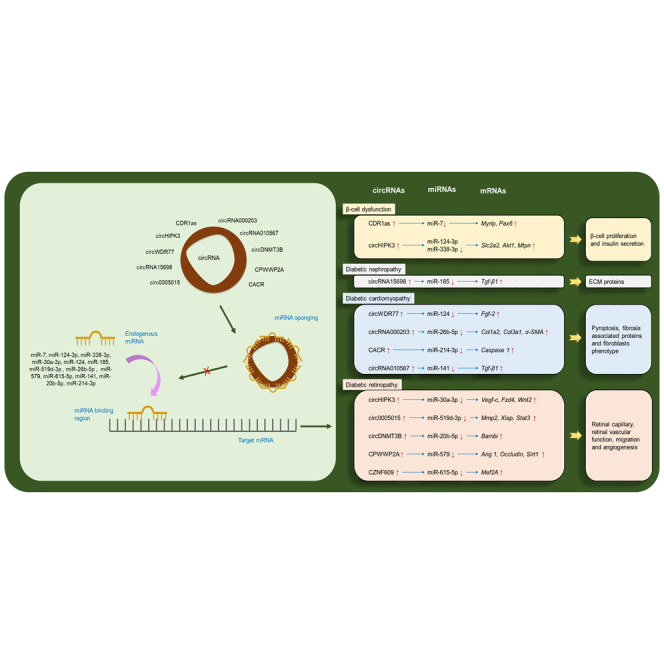

## Teaser

Circular RNAs (circRNAs) are closed long noncoding RNAs, and have received increasing attention in disease biology. This review highlights the circRNA-miRNA-mRNA regulatory axis in diabetes and its associated disorders. More than 20,000 novel circRNAs have been identified to date, although only a few are identified with their regulatory pathways or interaction networks.

## Introduction

Diabetes mellitus (DM) is a rapidly growing epidemic and a leading metabolic and endocrine disorder caused by chronic hyperglycemia resulting from insulin resistance.^1^ It is estimated that 463 million people have diabetes, and this number is expected to reach 578 million by 2030 and 700 million by 2045.^2^ Insulin resistance and endothelial dysfunction are the two major factors that impede other vascular complications in patients with diabetes.^3^ Imbalanced secretion of endothelium-derived growth factors results in end-organ damage.^4^ Other than insulin resistance, factors like the release of free fatty acids and lipid toxicity, oxidative stress, and dyslipidemia also contribute to the impairment of endothelium.^5^ In diabetic patients, vascular complications such as nephropathy, retinopathy, and cardiomyopathy presents serious manifestations with poor life expectancy.^6^ The body is exposed to agents that produce reactive oxygen species (ROS) by transferring free unpaired electrons, causing cellular component oxidation. As a defense, the body obtains exogenous antioxidants from the diet that can neutralize these species. The imbalance between ROS and antioxidants leads to oxidative stress, creating pathological conditions like diabetes.^7^ Studies have also identified the play of epigenetic regulators in the pathogenesis of diabetes.

In recent times, the advent of new sequencing technologies has helped understand the transcription of human genome into RNAs, among which only 1–2% were found to code for a protein, and the rest do not.^8^ These noncoding RNA (ncRNA) transcripts that do not code for a protein were referred to as “junk” for a long time and later were described as a highly conserved functional molecule that regulates the gene expression in various manners.^9^ Based on the size of ncRNAs, they have been broadly classified as small noncoding RNAs (sncRNA) and long noncoding RNAs (lncRNAs). The transcripts such as microRNA (miRNA), small interfering RNAs (siRNAs), and piwi-interacting RNAs (piRNAs) are below 200 nucleotides (nts) and are referred to as sncRNAs, whereas the lncRNAs above 200 nts include promoter-associated transcripts (PATs), enhancer RNAs (eRNAs), and circular RNAs (circRNAs).^10^ Recently, the circular lncRNAs or the circRNAs have gained focus among researchers and are extensively studied for their regulatory role in cellular signaling.^11^ Although circRNAs were unnoticed in the previous decades, advances in genomic sequencing, transcriptional profiling, computational tools, and structural biology have made us understand their importance in the pathogenesis of various diseases, including diabetes. Researchers have identified the functionality of circRNA to sponge its endogenous pair, the miRNA, and thereby play an important role in the progression of diabetes and its complications. For example, Zhou et al. identified a novel circRNA, circRNA 010567, that targets and increases the expression of *TGF-β1* by sponging miR-141, thereby promoting myocardial fibrosis.^12^ In this way, this review article will summarize the findings of the circRNA-miRNA-mRNA interaction network in diabetes and its associated complications.

## Regulatory functions of circRNA and miRNA

### Biogenesis of circRNA

In the mid-1970s, circRNAs were discovered as viroids in RNA viruses and assumed to be an error in processing the RNA splicing mechanism. circRNAs were reported by Hsu et al. in the cytoplasm of mammalian cells using electron microscopy.^13^ The circRNAs are single-stranded RNA molecules that differ from their linear RNA form by forming a continuous loop covalently joined between their 5′ and 3′ ends.^14^ The circRNAs are formed by a back-splicing mechanism wherein the 5′ terminus of the upstream pre-mRNA is non-collinearly spliced to the downstream exons of the 3′ terminus.^15^

**Figure 1:**
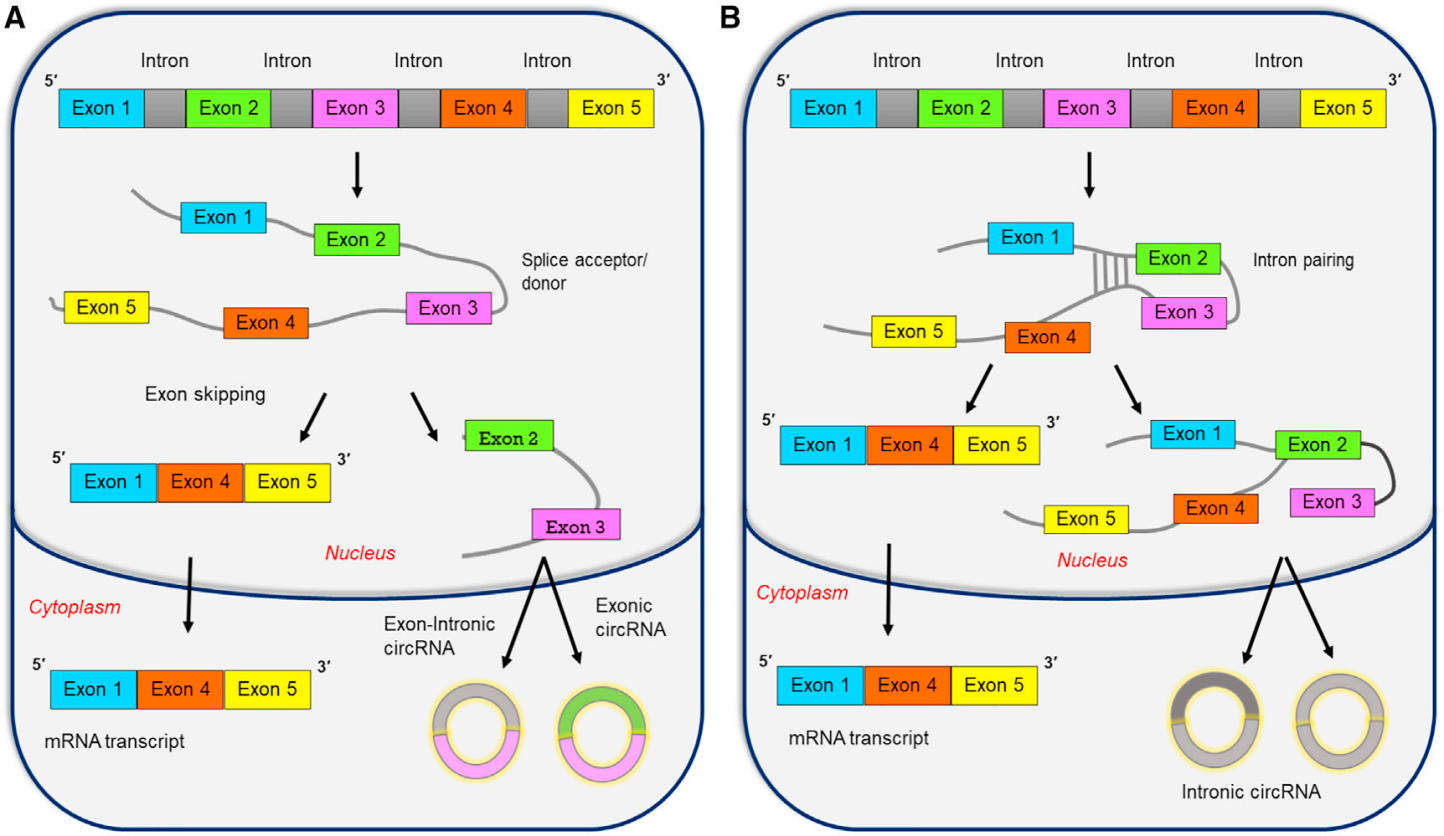
Biogenesis of circRNA (A) In lariat-driven circularization, the formation of exonic circRNA is through exon skipping guided by a splice acceptor/donor. (B) In intron pairing-driven circularization, the formation of intronic or exon-intronic circRNA is by the hybridization of flanking introns with close proximity and is independent of exon skipping and brings the splice site.

Jeck et al. (2010) have proposed two models for exon circularization: the lariat-driven circularization models and the intron pairing-driven model.^16^ In lariat-driven circularization (Figure 1A), two transcripts are produced due to the splicing of pre-mRNA.^17^ These two transcripts are mRNA that lacks skipped exons and lariat containing those skipped exons, thus creating a chance for circularization. Splicing of exon lariat results in the formation of circRNA and an intron lariat. These introns can form lasso shaped structures, but they tend to degrade due to branching enzymes.^18^ In intron pairing-driven circularization (Figure 1B), exons of the circRNA are complementary to introns flanking them for binding to each other. Splice sites with close proximity come close to each other, increasing the possibility of back splicing.

The circRNA contains up to five exons, and the introns bordering them are three times longer than their linear form. Research has revealed many complementary Alu repeats in the intronic region, which accelerates the splice site to locate each other and promote circularization easily.^16^ Due to their closed-loop structure, they lack 5′ and 3′ regions containing a poly-A tail and cap region, making them resistant to exonucleases; e.g., ribonuclease R (RNase R) mediated cleavage.^19^ Eunuka et al. reported that, due to this, circRNA lasts 2.5 times longer compared with their linear RNA form.^20^ RNase R degradation can accurately select the closed-loop nature of circRNA over their linear forms as they can degrade linear RNA and its poly-A tail. circRNAs are resistant to RNA degradation as they lack a 5′ cap and 3′ tail compared with their linear forms.^21^

The formation of circRNA is facilitated by reverse binding Alu elements to RNA helicases, DExH-Box Helicase 9 (DHX9) and harboring inverted repeats of long introns, which flanks the genomic structure of long exons.^22^ In the normal growing cells, the formation of circRNA by back-splicing event occurs due to NF90/NF110 binding to A/U-rich elements in the intronic region.^23^ Back splicing for circRNA is also promoted by heterogeneous nuclear ribonucleoprotein L (HNRNPL),^24^ muscle bind (MBL),^25^ and RNA-binding protein quaking I (QKI).^26^ It has been reported that MBL upgrades circMBL back splicing by binding to pre-mRNA and decreasing the levels of MBL, whereas QKl brings two cyclic sites closer by binding to both flanking ends of introns and combining the cyclic exons.^25^

Production of circRNA can also be regulated by a RNA-binding protein, fused in sarcoma (FUS),^27^ to the intron flanking region in back-splicing junctions, which can further be controlled by heterogeneous nuclear ribonucleoprotein (hnRNP) and serine-arginine (SR) proteins.^28^ RNA-editing enzymes such as adenosine deaminases acting on RNA (ADAR) can exterminate double-strand chain interaction and bind to flanking intron's double-stranded areas to inhibit circRNA formation.^29^ Recent studies have indicated increased circRNA synthesis by inhibiting the pre-mRNA processing mechanisms such as spliceosomes by extension of read-through of the downstream genes.^30^

Based on the sequence and domains of circRNA, they have been categorized as follows: (1) intronic circRNA (ciRNA), which are located in the nucleus and are joined by a 2′–5′ phosphodiester bond. This class of circRNAs features the enrichment of 3′ branch site containing an 11 C motif and a 5′ splice site containing 7 GU motif.^15^ (2) Exonic circRNAs (ecRNAs), which are mostly identified as an miRNA sponge, and are located in the cytoplasm. Besides being involving in gene transcription through sponging miRNAs, they also interact with RNA-binding proteins and participate in protein translation. This type of circRNA is formed by exon skipping and is joined by 3′–5′ phosphodiester bond.^31^ (3) Exon-intron circRNA (ElciRNA), which is also formed by exon skipping and located in the nucleus. The ends of this type of circRNA are joined by a 3′–5′ phosphodiester bond and bind with RNA to promote transcription of target genes.^32^

Liu et al. identified the ability of endonuclease RNase L to degrade the circRNA upon viral infection or poly (I:C) stimulation. As they lack 3′ and 5′ terminus, endoribonucleolytic cleavage was also reported to occur.^33^ Kim et al. identified the role of N6-Methyladenosine (m6A) methylation in the degradation of circRNAs by downregulating YTH N6-methyladenosine RNA-Binding Protein 2 (YTHDF2) through RNase P/MRp complex.^34^ A brief on the basic characteristics of circRNA is given in Table 1.

**Table 1:** Basic characteristics of circular RNA

RNA type	Noncoding^10^
Splicing mechanism	back-splicing^15^
Degradation	resistant to exonuclease cleavage^19^
Exon and intron length	exons are in the range 1–5, while introns that flank them are three times longer compared with linear counterparts^16^
Structure	closed loop^35^
**Types of circRNA**	
Exonic circRNA	located in the cytoplasm and joined by 3′–5′ phosphodiester bond; exonic circRNA functions as miRNA sponge, may also function as a sponge for RBPs^15^
Intronic circRNA	located in the nucleus and joined by 2′–5′ phosphodiester bond; intronic circRNA functions in regulating gene expression^36^
Exon-intron circRNA	located in the nucleus and joined by 3′–5′ phosphodiester bond; exo-intron circRNA functions in regulating gene expression^32^

RBPs, RNA-binding proteins.

### Functions of circRNA

A diversified set of circRNAs have been identified for their functions like sponging, acting as decoys, or translatable elements that alter the gene or protein expression. (1) circRNAs can modulate miRNA activity by functioning as a sponge.^37^ A single circRNA can bind with one or many miRNAs with its circular sequence. A well-studied example of miRNA sponging is circRNA-CDR1as, which was found to harbor 63 binding sites for miR-7.^38^ (2) By interacting with proteins, circRNAs were found to function as decoys and alter the cellular function. Studies have demonstrated that CDK2/p21 and HIF-1α/ID1 get trapped by circFOXO3 in the cytoplasm, which is shown to block cell cycle progression and induce senescence.^39^ The silencing of circFOXO3 enhanced cell viability, whereas it led to apoptosis when they were ectopically expressed. Ectopic expression of circFOXO3 was reported to repress the p53 levels and increase the protein level of FOXO3. p53 levels decrease when circFOXO3 leads to MDM2-induced p53 ubiquitination, followed by degradation. circFOXO3 prevents FOXO3 ubiquitination and degradation caused by MDM2 due to its low binding affinity with FOXO3 protein.^40^ (3) circRNAs are a type of lncRNAs having a low potential protein-coding ability. They also contain m6A modifications or internal ribosome entry sites (IRESs) and can be translated into peptides.^41^ Pamudurti et al. have observed a cap-independent translation *in vitro* and *in vivo* using the ribo-circRNAs UTR. It was also reported that the circMBL isoform is likely to be regulated by FOXO. These studies provide evidences that circRNAs can also be translated.^42^

**Figure 2:**
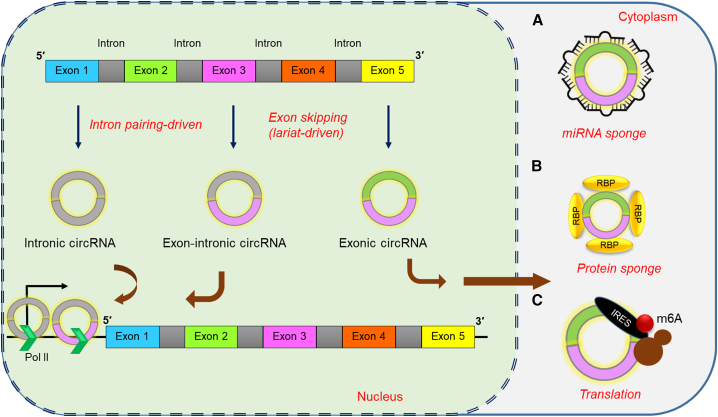
Functions of circRNA The intronic and exon-intronic circRNAs act on the promotor region to recruit Pol II, thereby promoting transcription of target genes. The exonic circRNA that is exported to the cytoplasm functions as an miRNA sponge and sequesters the endogenous miRNA that inhibits target mRNA (A), or binds with RNA-binding proteins and act as a protein sponge and mediates their action (B). m6A and IRES modification can promote circRNA to translate to a protein (C). RBP, RNA-binding protein.

Dysregulated cellular functions by circular RNAs have been reported with different diseases. For example, fibroblast proliferation and migration can also be stimulated by activating alveolar macrophages, which are promoted by circZC3H4^43^ and circHECTD1.^44^ Disruption of certain circRNAs, such as circHIPK2^45^ and circHECTD1,^46^ can inhibit the activation of astrocytes, which may benefit stroke recovery. Some circRNAs have also been reported to regulate apoptosis.^47^ For example, when Itchy E3 ubiquitin protein ligase (ITCH) was increased in non-small cell lung cancer (NSCLC) cells by has_circ_0043256, it was observed to induce apoptosis, while an increase of ERBB2 in the nucleus pulposus (NP) cell by circGRB10 was reported to inhibit apoptosis.^48^ CDR1as have been found to have regulatory roles in specialized β cells of pancreatic islets that produce insulin. It was inferred that CDR1as might inhibit the function of miR-7 in islet cells, thus improving insulin secretion.^49^ The diversified functions of circRNA are depicted in Figure 2.

### Biogenesis and functions of miRNA

miRNAs are one of the classes of sncRNAs, typically 20–23 nt in length. They have been reported to alter mRNA and protein expressions by regulating their transcriptional and post-transcriptional levels.^50^ Among all the identified miRNAs to date, about half of them are processed more from introns compared with exons of protein-coding genes; i.e., they are intragenic,^51^ while regulation of other miRNAs is controlled by their promoters and is independently transcribed of host gene; i.e., they are intergenic.^52^ miRNAs can be transcribed into clusters as well.^53^

The majority of mature miRNAs are transcribed from pri-miRNA processing with the help of a microprocessor complex consisting of Drosha (ribonuclease III enzymes) and an RNA-binding protein, DiGeorge Syndrome Critical Region 8 (DGCR8) in the nucleus.^54^ DGCR8 recognizes the m6A GGAC region in the pri-miRNA motif, which is then cleaved by Drosha, leading to 3′ overhang. The pre-miRNA, which is generated from 3′ overhang, is exported to the cytoplasm through exportin 5 (XPO5)/RanGTP complex.^55^ The miRNA duplex is then loaded onto the argonaute (AGO) in an ATP-dependent manner, generating the guide and passenger strands based on the degree of complementarity.^56^ The guide strand and the miRNA response element form the miRNA-induced silencing complex (miRISC), which induces AGO2 endonuclease activity to cleave the target mRNA site.^57^^,^^58^

Some miRNAs prefer Drosha/DGCR8, an independent pathway to produce pre-miRNAs from the introns of mRNA during splicing and debranching (e.g., mitrons).^59^^,^^60^ This type of processed miRNAs contains 3′ overhang produced by terminal uridylyltransferase (TUTase), which undergoes monouridylation for efficient dicer processing. These pre-miRNAs are then exported to the cytoplasm through exportin 1. The shorter miRNAs or endogenous short hairpin RNAs (shRNAs) are exported via exportin 5 to the cytoplasm after being recognized and cleaved by Drosha/DGCR8 complexes. These shorter transcripts are then loaded by AGO2 in the cytoplasm and turn into mature miRNA by poly(A)-specific ribonuclease (PARN)-mediated 3′–5′ trimming of 5p strand.^61^

## circRNA-miRNA-mRNA interaction in diabetes and associated complications

miRNAs are recorded to play a significant role in the pathogenesis of diabetes by controlling the upregulation or downregulation of the genes involved in signaling cascade.^62^ For example, miR375 is involved in the development of pancreatic β cells. Studies have found the altered expression of this particular miRNA in T2DM patients alters glucose homeostasis by decreasing insulin secretion.^63^^,^^64^ Characterizing miRNAs based on their altered expression in diabetes and its associated complications has emerged from experimental studies with the aid of *in silico* databases. Several hyperglycemia-induced miRNAs, such as miR21, 192, 216, 217, and 377, are linked with the pathogenesis of diabetes and its complications. Therapeutic interventions of miRNA can inhibit the function of miRNA or repair its diminished activity. Experimentally, gene knockouts and antisense oligonucleotides have been widely used to inhibit the activity of miRNA, whereas replacing the function of miRNA can be achieved by introducing an miRNA mimic. Although the development of the delivery system for miRNAs to reach specific cells is complicated, an increasing number of patents have been applied for recently for miRNA-based therapeutics, especially in cancer. In this connection, BalkrishenBhat et al. have filed a patent targeting miRNAs for metabolic disorders through Regulus Therapeutics (US 20,180,171,334 A1). Many modifications for the delivery of miRNA into the muscle to reach a specific target are being carried out. For example, a 2′-OMe-modified anti-miR with phosphorothioate linkage for miR-103/107 to improve glucose homeostasis has been reported by Trajkovski et al.^65^ The miRNA-based therapeutics for clinical use in diabetic patients are still in the pipeline.

Besides regulating their targets, miRNAs were found to be controlled by several intrinsic factors, and circRNAs are one of them. circRNAs partly inhibit the miRNA activity by interacting with them, suggesting their possible role in gene and protein expression.^66^

**Figure 3:**
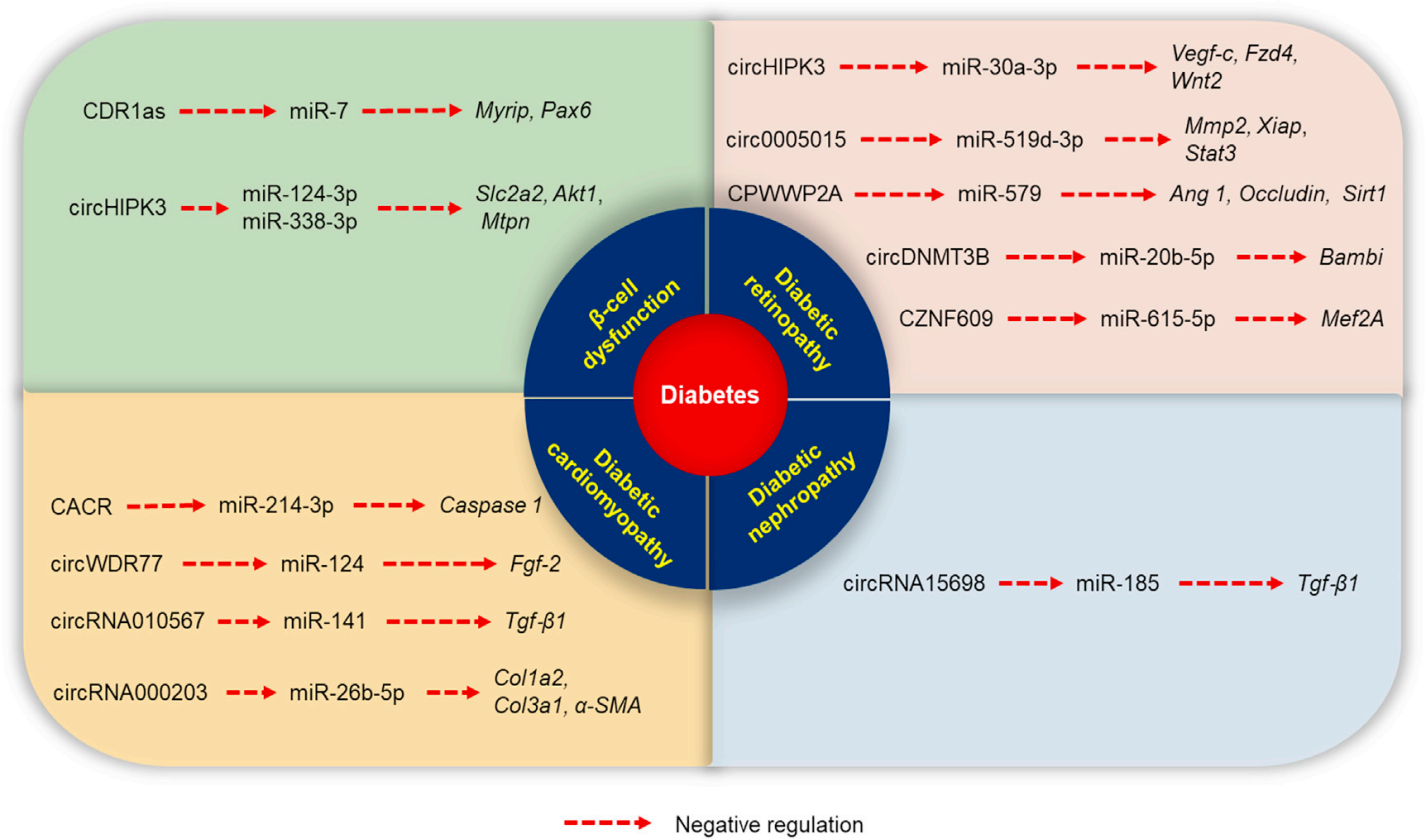
circRNA-miRNA-mRNA interaction in diabetes and its complications The circRNAs negatively regulate the miRNA, which inhibits the expression of mRNA.

circRNAs play a role in gene expression as they can partly inhibit miRNA activity. They bind to miRNA like a sponge and act as competing endogenous RNA, regulating the function of the target miRNA, thus indirectly targeting mRNA levels.^67^ The next section describes in detail the role of this interactive network in regulating diabetes and its associated complications (Figure 3). Table 2 represents the overview of this interaction network.

**Table 2:** circRNA-miRNA-mRNA interaction network

Name of the circular RNA	Target miRNA	Target mRNA	Study model	Diabetic complication	Outcome	Reference
CDR1as ↑	miR-7↓	*Myrip* and *Pax6* ↑	MIN6 cells (rat islet cells)	diabetes	affected insulin secretion	^49^
circHIPK3 ↑	miR-124-3p and miR-338-3p↓	*Slc2a2*, *Akt1*, and *Mtpn* ↑	human islets, MIN6B1 cells (rat islet cells)	diabetes	regulated essential β cell activities, altered expression in diabetes models	^68^
circRNA15698 ↑	miR-185 ↓	*Tgf-β1* ↑	diabetic mice C57BL/KsJ-db/db	diabetic nephropathy	promoted ECM-related protein synthesis	^69^
circWDR77 ↑	miR-124 ↓	*Fgf-2* ↑	human VSMCs and HEK 293T cells	diabetic cardiomyopathy	suppressed VSMC proliferation and migration	^70^
circRNA000203 ↑	miR-26b-5p ↓	*Col1a2*, *Col3a1*, and *α-SMA* ↑	diabetic db/db mice, C57BL/6 mice	diabetic cardiomyopathy	the enhanced fibrotic phenotype in cardiac fibroblasts	^71^
CACR ↑	miR-214-3p ↓	*Caspase 1* ↑	human cardiomyocyte AC16 cells	diabetic cardiomyopathy	alleviated pyroptosis in cardiomyocytes	^72^
circRNA010567 ↑	miR-141 ↓	*Tgf-β1* ↑	diabetic db/db mice, C57BL/6 mice	diabetic cardiomyopathy	suppressed fibrosis-associated protein resection in CFs	^12^
circHIPK3 ↑	miR-30a-3p ↓	*Vegf-c*, *Fzd4*, and *Wnt2* ↑	C57BL/6 mice	DR	altered retinal endothelial cell function and microvascular dysfunction	^73^
circ0005015 ↑	miR-519d-3p ↓	*Mmp2*, *Xiap* and *Stat3* ↑	HRVECs	DR	facilitated retinal endothelial angiogenic function	^74^
circDNMT3B ↓	miR-20b-5p ↑	*Bambi* ↓	Sprague-Dawley rats, HRMECs	DR	reduced retinal acellular capillary number and alleviated visual damage	^75^
CPWWP2A ↑	miR-579 ↓	*Ang 1*, *Occludin*, and *Sirt1* ↑	C57BL/6 mice, HRVECs and human retinal pericytes	DR	affected retinal vascular dysfunction	^76^
CZNF609 ↑	miR-615-5p ↓	*Mef2A* ↑	**Human umbilical vein endothelial cells (**HUVECs), C57BL/6 mice	DR	increased the migration and tube formation ability of HUVECs, and rescued HUVECs from oxidative stress or hypoxia-induced apoptosis	^77^

### CDR1as-miR7 interaction in regulating insulin homeostasis

Insulin homeostasis is essential to bridge the balance between glucose and insulin. As per the World Health Organization (WHO), more than 420 million adults live with diabetes, and most of them do not have insulin homeostasis.^78^ *Pax6* is one of the genes responsible for insulin production in β cells of pancreatic islets.^79^ In this connection, CDR1as (ciRS-7) has been reported as the target of *Pax6* mRNA and one of the most promising miRNA targets among several human circRNAs consisting of 71 binding sites or 26 clusters corresponding to miR-7.^80^ Hansen et al. demonstrated the regulatory function of CDR1as (ciRS-7) as an miR-7 sponge whose overexpression induced midbrain developmental defects in zebrafish. This phenotype also reported the alteration in the expression of miR-7 in the central nervous system.^81^^,^^82^ Dysregulated expression of miR-7 has been proved with the loss of insulin secretion and insulin exocytosis, resulting in diabetes.^83^^,^^84^ Xu et al. reported the increased expression of insulin corresponding with overexpression of CDR1as, as demonstrated in min-6 and mouse islet cells. Further, it was also demonstrated that forskolin (labdane diterpene) induced CDR1as overexpression and phorbol myristate acetate (PMA) induced miR-7 downregulation, whereas only glucose was observed to regulate neither CDR1as nor miR-7.^49^ This study has reported that CDR1as overexpression with increased mRNA expressions of insulin and *Pax6* (known for increasing insulin secretion by binding to promoters of insulin gene). This study has also shown the decreased mRNA expression of *Myrip*, which is involved in insulin exocytosis, forming CDR1as-miR7-*Pax6*/*Myrip* interaction network in regulating insulin homeostasis.^49^

### circHIPK3-miR-124-3p and miR-338-3p interaction in β cell function

The main function of β-cells is to secrete insulin, thereby regulate glucose homeostasis in the body. At the time of digestion and where there is an increased availability of glucose, insulin starts to secrete from β-cells of pancreatic islets. The mRNAs, such as *Akt*, *Mtpn*, and *Slc2a2*, were reported to have a strong association with insulin secretion. circHIPK3, which targets the above-mentioned mRNAs, is one of the abundantly expressed circRNAs in the pancreatic islets that originates from the Hipk3 gene and is reported to reduce expression in the patients’ islets.^85^ Stoll et al. confirmed that circHIPK3 controls insulin secretion and has activity on glucose conversion in β-cells of db/db mice. The expression of mRNAs such as *Akt*, *Mtpn*, and *Slc2a2* was found to be downregulated upon inhibiting circHIPK3. Further, the authors also confirmed a drop in the luciferase activity of the construct containing the 3′ UTR of *Mtpn* upon silencing this particular circRNA. Using computational tools, miR-124-3p and miR-338-3p were found to target circHIPK3 involved in β-cell function by controlling insulin secretion and β-cell proliferation, respectively.^68^

### circRNAWDR77-miR124 interaction in atherosclerosis

High blood glucose levels in patients with diabetes are more susceptible to proliferation and migration of vascular smooth muscle cells (VSMCs), ultimately ending up in atherosclerosis.^86^ In 2017, Chen et al. proved the involvement of circRNA in regulating atherosclerosis. This study identified the differentially expressed circRNAs through microarray analysis and reported circWDR77 to be largely expressed in diabetic patients. Through bioinformatic analysis, the interaction between circRNA WDR77 and miR-124 was predicted and confirmed with qPCR. The expression of miR-124 was observed to be decreased in VSMCs treated with high glucose. Further, the mRNA *Fgf2* was also predicted to be a target of miR-124, which was then confirmed using a luciferase reporter assay. This study also proved the loop with circRNA and *Fgf2* mRNA by silencing circRNA WDR77, which inhibited the expression of *Fgf2*.^70^

### circRNA15698-miR185 interaction in diabetic nephropathy

Diabetic nephropathy (DN) is characterized by the proliferation of mesangial cells (MCs) and accumulation of extracellular matrix (ECM).^87^When exposed to hyperglycemic conditions, the expression of inflammatory cytokines in MCs is reported to progressively increase, which contributes to the progression of chronic kidney diseases like fibrosis.^70^ Hu et al. (2019) demonstrated the upregulation of circRNA15698 expression in total RNA extracted from the kidney cortex tissue of a db/db mice model. Further, upon knockdown of this particular circRNA, a reduction in ECM accumulation along with a decreased expression of fibrosis-related proteins in MCs were observed. The interaction of miR-185 with this circRNA was analyzed through a bioinformatics approach and validation using a luciferase reporter assay. This study also confirmed a reduced expression of miR-185 in mice models. The relation between circRNA and miRNA was examined upon knockdown of circRNA, which significantly increased miRNA level, suggesting a negative role. *Tgf-β1* was chosen as a target mRNA in this interactive network through bioinformatics studies and validated with luciferase reporter assay. Together, the results of this study have confirmed the role of the circRNA15698-miR185-*Tgf-β1* interaction network in the regulation of DN.^69^

### circRNA000203-miR-26b-5p interaction in diabetic cardiomyopathy

One of the risks of diabetes, diabetic cardiomyopathy (DCM) is attributed to insulin resistance, hyperglycemia, increased fatty acids, and myocardial fibrosis.^88^ Tang et al. (2017) demonstrated the upregulation of circRNA000203 and its parental genes in the myocardium of diabetic mice (db/db) compared with non-diabetic mice (db/m). The study also demonstrated that circRNA000203 increases the expression of *Col1a2*, *Col3a1*, and *α-SMA* in mouse cardiac fibroblasts induced by Ang-II. This particular circRNA was screened to bind with miR-26b-5p through bioinformatics tools and was analyzed to suppress the effects of Col1a2 and CTGF in cardiac fibroblasts. This study presented that circRNA000203 suppressed the interaction between miR-26b-5p and *Col1a2* and *Ctgf*. This leads to impaired miR-26b-5p inhibition effects on the fibrosis-associated genes *Col1a2*, *Col3a1*, *α-SMA*, and *Ctgf*, in cardiac fibroblasts. The results of this study together highlighted the role of circRNA000203-miR-26b-5p-*Col1a2/Col3a1/α-SMA* interaction network in the regulation of DCM.^71^

### CACR-miR-214-3p interaction in diabetic cardiomyopathy

The expression of caspase-1-associated circRNA (CACR) has been reported to be increased in high-glucose-induced cardiomyocytes. The authors of this study induced diabetic conditions by exposing the cells to high glucose condition and confirmed the activation of pyroptosis with increased mRNA and protein expression of *Nlrp3*, *Caspase-1*, and *IL-1β*. As per the previous literature reports, miR-214-3p was found to be one of the targets of *Caspase-1* and was confirmed to be reduced under high glucose conditions. Through a computational approach, CACR was predicted to bind with miR-214-3p. Hence the expression of CACR was inhibited using an Antisense Oligonucleotide (ASO), confirmed using western blotting. Further, silencing CACR was found to alleviate high-glucose-induced pyroptosis by increasing miR-214-3p and subsequently inhibiting its endogenous target, *Caspase-1*. The expressions of *Nlrp3*, and *IL-1β* were also low on silencing CACR but did not have many changes on silencing miR-214-3p, unlike *Caspase-1*. So, this study in detail demonstrated that CACR regulated pyroptosis through miR-214-3p/*Caspase-1* pathway in high-glucose-induced cardiomyocytes, suggesting its plausible role.^72^

### circRNA 010567-miR-141 interaction in myocardial fibrosis

A study conducted by Zhou et al. in 2017 has identified the circRNA010567 as one of the differentially expressed circRNAs in db/db mouse myocardium using microarray analysis. This study reported the expression of circRNA010567 to be significantly high among the other upregulated circRNAs. RNAi-mediated knockdown of this specific circRNA partly affected the expression of miR-141 in mouse cardiac fibroblast cells. A decreased expression of miR-141 was observed in the db/db mice myocardium, suggesting a negative regulation. Further, this study had found a higher affinity of *Tgf-β1* mRNA with miR-141 using bioinformatics, which was then confirmed with dual-luciferase reporter assays. Further, miR-141 was observed to increase the expression of *TGF-β1* significantly. Diabetic mice myocardial fibrosis is thus mediated by the circRNA010567-miR-141-*Tgf-β1* axis and thus was proposed as a novel target.^12^

### circHIPK3-miR-30a-3p interaction in retinal dysfunction

Diabetic retinopathy (DR) or retinal dysfunction is characterized by vascular permeability, capillary occlusion at the early stage (non-proliferative DR), and neovascularization at the later stage (proliferative DR).^89^ Although most of the cases have been reported with aberrant angiogenesis, the less appreciated contact of retinal dysfunction in diabetic patients is the impaired angiogenesis.^90^ In this context, Shan et al. have deciphered the increased expression of circHIPK3 in mouse retinal endothelial cells and human retinal vascular endothelial cells (HRVECs). This increase in circHIPK3 expression was observed to sponge the endogenous miR-30, which was confirmed using a luciferase reporter and fluorescence *in situ* hybridization (FISH) techniques. Angiogenic markers such as *Vegf-c*, *Fzd4*, and *Wnt2* were the potential targets and seemed to be reduced on silencing circHIPK3 with increased miR-30a-3p expression. This study has demonstrated the role of circHIPK3 in DR by inhibiting miR-30a, thereby increasing the target angiogenic genes’ expression and promoting angiogenesis.^73^

### circRNA0005015-miR-519d-3p interaction in DR

DR is characterized by increased vascular permeability and the growth of new blood vessels in the retina and posterior surface of the vitreous,^91^ which results in neurodegeneration and dysfunction of the microvascular retina.^92^ Retinal vasculature is characterized by the migration and proliferation of endothelial cells, which are largely affected by hyperglycemia.^93^ circRNA expression profiling carried out by Zhang and his research group in 2017 have identified 365 circRNAs to be upregulated in diabetic human retinas of among 529 differentially expressed ones. They have also identified an upregulated expression of circ0005015 from *Has2* gene locus in the plasma, vitreous, and preretinal fibrovascular samples among DR patients. Through bioinformatics tools, the regulatory expression of circ0005015 with miR-519d-3p was identified and was confirmed using a luciferase reporter assay. This study also demonstrated the decreased expression of *Mmp2*, *Stat3*, and *Xiap* genes in HRVECs upon overexpression of miR-519d-3p, and the ability of tube formation was decreased. This study has suggested the existence of the circRNA0005015-miR-519d-3p-*Mmp2/Stat3/Xiap* interaction network in DR.^74^

### circRNA DNMT3B - miR-20b-5p interaction in DR

Recently, the role of circRNA DNMT3B has been demonstrated by Zhu et al. (2019) in high-glucose-induced human retinal microvascular endothelial cells (HRMECs). As per previous literature reports, the expression of miR-20b-5p was examined in the retina of diabetic rats. The study showed an increased expression of miR-20b-5p in the retina of diabetic rats compared with the control ones. Further, this was validated *in vitro* in high-glucose-induced HRMECs. Using the bioinformatics approach and as per the previous reports, the research team further investigated the effect of *Bambi* in high-glucose conditions.

Also, silencing *Bambi* increased proliferation, migration, and tube formation of HRMECs and counteracted the effects caused by inhibiting miR-20b-5p. The expression of circRNA DNMT3B was decreased and was in line with fibrovascular membranes of DR patients. Using the bioinformatics tools, this particular circRNA was found to sponge miR-20b-5p. This was validated *in vitro* and overexpression of circRNA DNMT3B attenuated high-glucose-induced effects of miR-20b-5p and *Bambi*. Hence, the results of this study showed that circRNA DNMT3B regulated high-glucose-induced HRMEC function by targeting miR-20b-5p and *Bambi*, suggesting its role in DR.^75^

### CPWWP2A-miR-579 interaction in DR

Dysregulated functions of retinal microvasculature that comprise endothelial cells and pericytes have been reported to be a major cause of vascular leakage in patients with DR.^94^ An elevated expression of cPWWP2A in pericytes was reported by Liu et al. in 2018, suggesting their role in DR. This study reported 89% similarity of circRNA0000254 with the human genome in db/db mice by performing microarray analysis, which was then named CPWWP2A as its host gene is PWWP2A. The exosome carrying this particular circRNA from endothelial cells to pericytes has been assessed. With bioinformatics aid, the interaction of cPWWP2A with miR-579 was confirmed using a luciferase reporter assay. Co-localization of circRNA with miRNA has been reported by the RNA-FISH hybridization technique. The expressions of target genes, such as Angiopoietin1 (*Ang1*), *Occludin*, and *Sirt1*, were found to be low upon overexpression of miR-579 and silencing CPWWP2A. This study witnessed the interaction network between cPWWP2A-miR-579-*Ang1/Occludin/Sirt1* in the regulation of vascular dysfunction in DR.^76^

### cZNF609- miR-615-5p interaction in DR

Vascular dysfunction, impaired angiogenesis, and vessel loss are the major hallmarks of ischemic progressions and are also persistent in DR.^95^ The C57BL/6 mice were exposed to a hypoxic environment with streptozotocin injection to make them retinopathic, and the regulation of cZNF609 was analyzed by Liu and his research group in 2017. The study revealed the cytoplasmic localization of cZNF609 and also explored the pro-angiogenic role of this particular circRNA on silencing cZNF609, which decreased retinal vessel loss and suppressed angiogenesis. Further, the inhibition and sequestration of miR-615-5p by cZNF609 to improve the *Mef2A* levels has been studied. This study has provided insight on cZNF609-miR-615-5p-*Mef2A* in the pathogenesis of vascular dysfunction.^77^

## Database for detecting circRNA and miRNA interaction with mRNA

**Figure 4:**
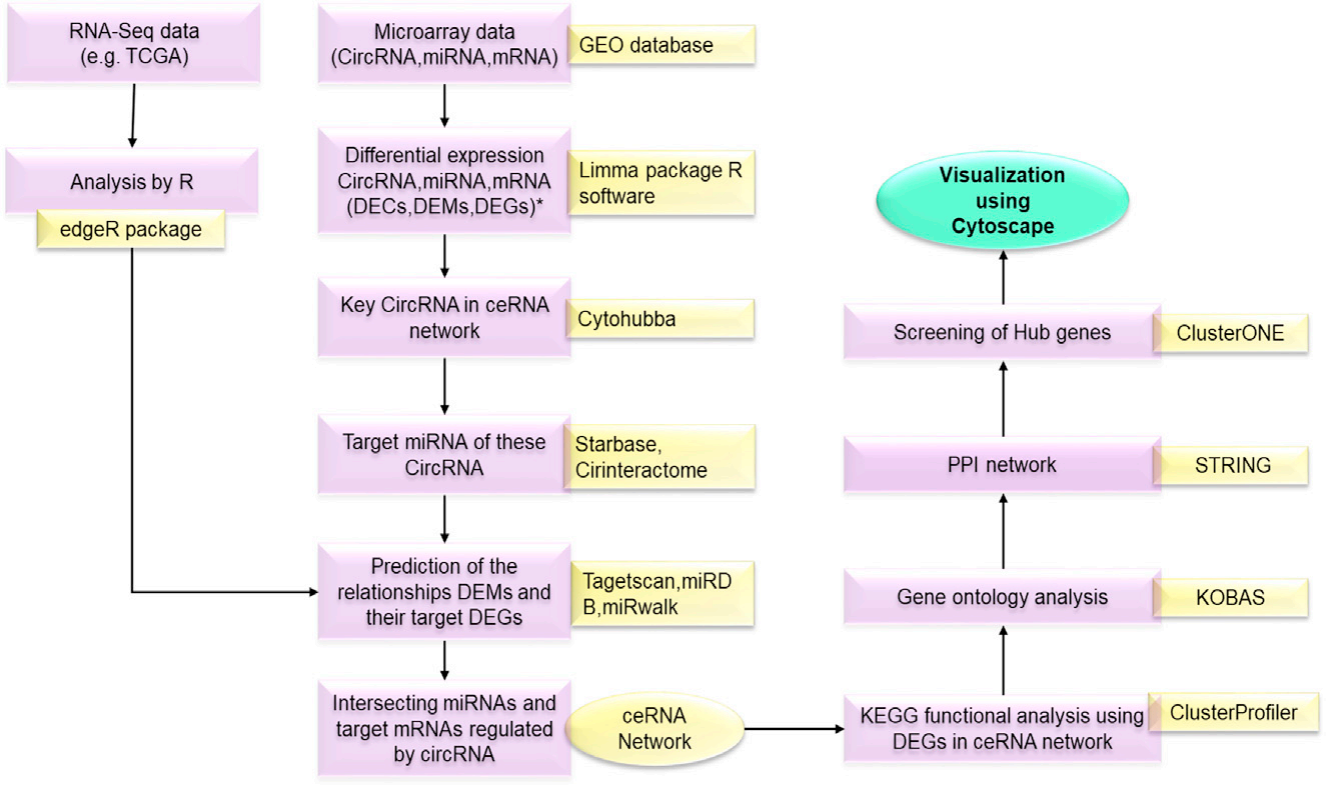
Construction of circRNA-miRNA-mRNA interaction network The step-wise construction of circRNA-miRNA-mRNA interaction network using bioinformatics tools. ∗Differentially expressed circRNAs (DECs), differentially expressed miRNAs (DEMs), and differentially expressed genes (DEGs).

To study the various aspects of circRNA and miRNA, multiple algorithms have been developed to detect both and their target interactions from the retrieved RNA sequences. These algorithms have helped develop various databases in which particular circRNAs or miRNAs can be explored with respect to their functions. Some of these are freely accessible for curating circRNAs or miRNAs from different species and providing more information about their role in various diseases.^96^ Tables 3 and 4 represent the available circRNA and miRNA databases to get information on their target interaction networks. A step-wise construction of this interaction network with the aid of bioinformatics tools is shown in Figure 4.

**Table 3:** Database for circRNA prediction

Online database	Remarks	Reference
circBase	annotation of circRNAs from the eukaryotic cell	^113^
starBase	contains information about different types of RNAs. This tool can be used for the detection of miRNA-circRNA interaction	^97^
circlncRNAnet	this tool aims to test *in silico* hypotheses of ncRNA-based functions by keeping a record of RNA sequencing data	^98^
circ2Traits	this online tool gives data about the positions of circRNAs in the genome and associated diseases	^99^
DeepBase	this tool provides a platform based on next-generation sequencing for annotation and discovery of ncRNAS	^31^
CircInteractome	this tool coordinates circRNA with RBPs	^100^
CirCpedia	this database gives information about various human and mouse samples through reverse splicing	^101^
circRNADb	this online tool gives information about more than 30,000 exons with circRNA in the human genome	^102^
TSCD	this tool helps in the characterization of tissue-specific circRNAs	^103^

**Table 4:** Database for miRNA prediction

Online database	Remarks	Reference
Target scan	searches for conserved 8mer and 7mer sites matching the seed region of each miRNA for predicting their biological targets	^104^
miRDB	online target prediction database; analyzes thousands of miRNAs and their targets through SVM learning machine by miRtarget	^105^
miRanda	identifies target genes and their level of downregulation at mRNA level	^106^
RNAhybrid	online prediction tool with unique features such as speed up of seed match, seed region G:U base pairing	^107^
MirGator	online portal based on deep sequencing and mRNA target prediction	^108^
miRecords	provides validates data regarding miRNA mRNA targets among seven animal species	^109^
miRTarBase	contains experimentally validated miRNA target interactions data	^110^

SVM, support vector machine.

## Discussion and future prospectus

In this review, we have analyzed the literature on the circRNA-miRNA-mRNA interaction network in diabetes and its associated complications. Recently, research in the field of noncoding RNAs has received increasing attention in disease biology. Of note, more studies focusing on the mechanism of miRNA sponge are being elucidated. The inhibitory effects of miRNAs have been revealed in many studies and are reported to be strongly associated with various diseases, including diabetes. Hence, exogenous tools like synthetic miRNA mimics and agomiRs that bind to the 3′ UTR to modify the expression of native genes have started to gain interest in the research community. In parallel, a few researchers also focus on the upstreams of miRNA to inhibit its expression. circRNA is one of them that mainly act as an miRNA or protein sponge. The use of circRNAs in therapeutic avenues has been reviewed by Holdt et al.^111^ The circRNAs can be synthesized or modified chemically as miRNA mimics using RNA ligases or ribozymes and introducing photolabile linkers. This could prevent the linearization and further inactivation of circRNAs. Further, chemical modifications to improve stability and binding affinity or coating circRNAs with proteins make the system easier to recognize. The delivery of any naked RNA and its half-life into any living system is, however, tricky. Research in the field of encapsulating RNAs into nanovesicles is ongoing.

The physiological functions of most of the circRNAs are yet to be identified, and may reveal some of their abilities to act as protein counterparts. Important identified circRNAs are exonic circRNAs that function as miRNA sponges to counteract and alleviate the miRNA-induced changes. The role of intronic circRNAs is less explored. Advances in RNA technologies would help us foresee many developments in circRNA research in the near future.

### Conclusion

The focus of research has been expanded in identifying several epigenetic tools involved in the pathology and progression of various diseases. This review has summarized the circRNA-miRNA-mRNA interaction network in diabetes and its associated disorders. More than 20,000 novel circRNAs have been identified to date,^112^ although only a few are identified with their regulatory pathways or interaction networks. The importance of identifying the interaction network between them is that it would shed light on the holistic and mechanistic picture of the regulation of various genes and their upstream elements in response to different pathological conditions. In the future, extensive research in this field would allow better diagnostics and provide more knowledge on the interactive mechanisms.
